# Supplementary material for: Paeoniflorin Inhibits Porcine Circovirus Type 2 Replication by Inhibiting Autophagy and Targeting AKT/mTOR Signaling
Source: Vet Sci. 2025 Feb 2;12(2):117. doi: 10.3390/vetsci12020117 (PMC11860941; doi:10.3390/vetsci12020117)
Supplement: Supplementary file 1 [file vetsci-12-00117-s001.zip › Supplementary Information.zip!/Supplementary Figures.pdf]

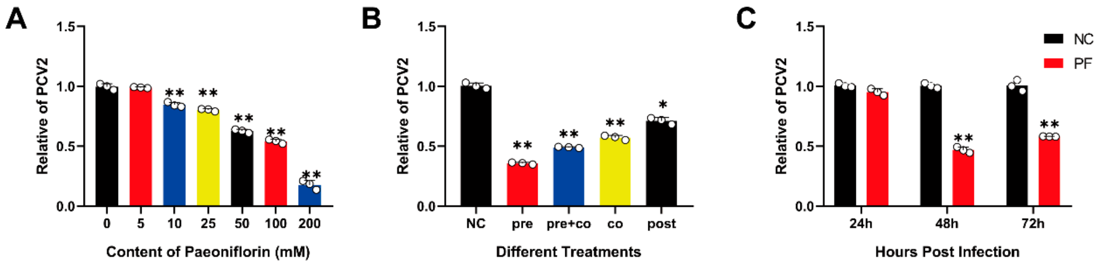

Figure S1 Measurement of the protein gray value in Figure 3.

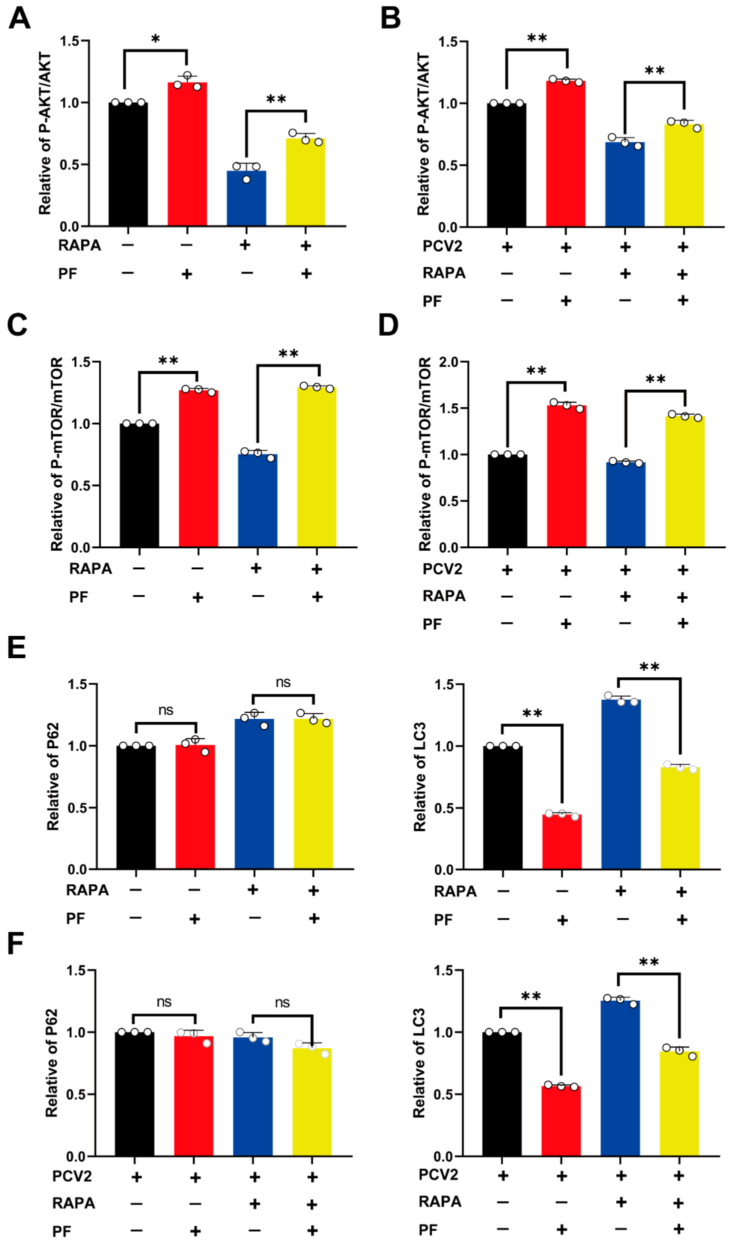

Figure S2 Measurement of the protein gray value in Figure 6.
